# Perceptions of Research Integrity Climate in Hungarian Universities: Results from A Survey among Academic Researchers

**DOI:** 10.1007/s11948-022-00382-5

**Published:** 2022-06-30

**Authors:** Anna Catharina Vieira Armond, Péter Kakuk

**Affiliations:** 1 Department of Behavioural Sciences, Faculty of Medicine, University of Debrecen, Debrecen, Hungary; 2 Department of Public Health and Epidemiology, University of Debrecen, Debrecen, Hungary; 3 Center for Ethics and Law in Biomedicine, Central European University, Budapest, Hungary

**Keywords:** Research integrity, Hungary, Organizational climate, Responsible conduct of research

## Abstract

Research integrity climate is an important factor that influences an individual’s behavior. A strong research integrity culture can lead to better research practices and responsible conduct of research (RCR). Therefore, investigations on organizational climate can be a valuable tool to identify the strengths and weaknesses of each group and develop targeted initiatives. This study aims to assess the perceptions on integrity climate in three universities in Hungary. A cross-sectional study was conducted with PhD students, postdocs, and professors from three Hungarian universities. The survey included demographic questions, such as gender, age, scientific field, academic rank, and the Survey of Organizational Research Climate (SOURCE). A total of 432 participants completed the survey. Our results show that postdocs and assistant professors perceived integrity climate more negatively than PhD students and full professors in every survey scale. Contrarily, PhD students perceive more positively than the other groups. Disciplinary differences show that researchers in the Biomedical sciences perceive regulatory bodies to be fairer when evaluating their projects than those in the Natural sciences. Natural sciences also perceive more negatively how the department values integrity when compared to Humanities. Humanities perceive more positively Advisor/Advisee Relations than Biomedical Sciences. Our results suggest that institutions should pay more attention to early career researchers, especially insecure and temporary positions like postdocs and assistant professors. They should provide RCR resources, socialize them in RCR, and set more reasonable expectations. Moreover, department leaders should develop initiatives to foster better integrity climates.

**Electronic supplementary material:**

The online version of this article (doi:10.1007/s11948-022-00382-5) contains supplementary material, which is available to authorized users.

## Introduction

There is a widely held concern that various forms of research misbehavior and breaches of research integrity are eroding the public trust in science and weakening the reliability of research results (Steneck, 2006). Besides the development of educational materials and guidance documents, research organizations responded with multiple research initiatives that attempt to understand the emergence and causes of research integrity breaches. Academic journals are dominated by discussions regarding prominent misconduct cases (falsification, fabrication, and plagiarism). Similarly, media reports usually focus on the violations of a particular scientist accused of misconduct (Armond et al., 2021). The initial focus of research integrity studies on misconduct and individual behavior has been slowly expanded with studying external, structural, and institutional factors in understanding a wider variety of questionable research practices (Martinson et al., 2005; Steneck, 2002, 2006). Interests in studying the organizational climate of researchers as external determinants of questionable research practices grow as these could also identify points of interventions for specific research institutions in fostering research integrity (Haven et al., 2019).

Organizational climate is “*the shared meaning organizational members attach to the events, policies, practices, and procedures they experience and the behaviors they see being rewarded, supported, and expected*” (Ehrhart et al., 2013). The term “organizational research climate” refers to the current patterns of organizational life and behavior related to research activities among organizational members and leaders (Schein, 1999). Organizational research climate studies focus on the institutional work environment of researchers, whether the departmental or university climate strengthens, supports, or erodes research integrity (Martinson et al., 2016; Mumford et al., 2007). The Survey of Organizational Research Climate (SOURCE) instrument has been developed to measure the organizational research integrity climate in academic research settings (Thrush et al., 2007).

The theoretical background of SOURCE originates in organizational justice as an umbrella term used to refer to individuals’ perceptions about the “fairness” of decision-making and resource distribution within organizations and the behavioral consequences of those perceptions (Martinson et al., 2010). If people perceive injustice in their organization, they are more likely to behave in ways that, in their mind, compensates for the perceived unfairness. In a research climate where perceived injustice is high, researchers would be expected to be more likely to engage in intentional research misconduct (falsification, fabrication, and plagiarism) or questionable research practices (Haven et al., 2019). The development of SOURCE has also been grounded in the Institute of Medicine and the National Research Council’s recommendations in 2002 on open systems conceptual framework for research integrity. It recognizes research integrity as an outcome of processes influenced by multiple factors (institution’s visible ethical leadership, socialization and communication processes, and the presence of policies, procedures, structures, and processes to deal with risks to integrity) (Martinson et al., 2013).

The SOURCE instrument was used in several recent studies in various contexts. Wells et al. studied three research intensive universities and found that researchers in different phases of their career perceive the research integrity climate differently (Wells et al., 2014). Doctoral students perceived the climate to be fairer compared to senior scientists in that scholarly integrity was valued. Senior scientists perceived more positively the resources for conducting research responsibly (Wells et al., 2014). This study also found a significant difference regarding SOURCE scores between different organizational subunits. The scores of some units were twice as negative compared to others or compared to the overall mean scores of the university. This indicates that research climate may vary significantly within institutions. One factor that accounts for these stark differences between subunits was the scientific field.

Another more recent study assessing research integrity climate focused on differences in academic rank and scientific field in two university medical centers and two universities in Amsterdam (Haven et al., 2019). The study by Haven et al. also showed differences regarding the research integrity climate perceptions of academic rank and scientific fields. Small fields like the Humanities perceive their department’s expectations as more negative compared to other scientific fields. According to this study, the natural sciences perceive the climate more positively. Associate and full professors perceive a more positive research integrity climate than assistant professors, postdocs, and PhD students.

These results show that the perceptions of integrity climate change across disciplines and career stages. Therefore, our study aims to determine how scientists experience the research integrity climate, stratified for academic rank and scientific field, in three large Hungarian universities with doctoral schools covering the full spectrum of disciplines. To our knowledge, this is the first study that investigates the research integrity climate in the country.

## Methods

This study was reviewed and approved by the Scientific and Research Committee of the Medical Research Council in Hungary (Approval number: IV8159-1/2020/EKU, date: 9.10.2020).

### Sample

All Doctoral Schools from the three largest comprehensive universities in Hungary (University of Debrecen, Pécs, and Szeged – 65 Doctoral Schools combined) were invited to participate. Of the total, sixteen Doctoral Schools agreed to participate in the study, with a total of 2557 registered PhD students, postdocs, and professors, according to the Hungarian Doctoral Council platform.

### Procedure

The Doctoral Council of each university was contacted to participate and asked to forward the invitation letter for each Doctoral School. The Doctoral Schools that agreed to participate were asked to send by email the letter along with the survey link for every registered researcher (PhD students, postdoctoral trainees, assistant professors, associate professors, and full professors). The Doctoral Schools were also asked to send two reminders, each one week apart. The data was collected between December 2020 and February 2021.

The survey was created using Qualtrics (Qualtrics, Provo, UT, USA). It consisted of the informed consent and demographic questions (gender, academic rank, age, and scientific field) and the Survey of Organizational Research Climate (SOURCE).

### Instrument

The Survey of Organizational Research Climate (SOURCE ©) is a validated questionnaire developed to assess the organizational climate of research integrity in an academic setting. The survey’s license was obtained, and the translation was authorized. The questionnaire includes 32 items, 28 items consisting of 7 subscales, two items assessing the global perception of the institutional environment, and two items of global perception of the department or program. The items are scored on a 5-point Likert scale (1 = ‘Not at all, 5 = ‘Completely’). A sixth option, “No basis for judging” is also offered not to force a response. The survey consists of two parts. The first part contains 11 questions on one’s perception of research climate in the wider institution. The second, with 21 items, regards one’s perception of research climate in the closer working environment, department, or program.

The 7 subscales assess the following areas: (1) Institutional RCR Resources (6 items). This scale assesses perceptions of the effectiveness of RCR resources and research-related communications, understanding of misconduct reporting procedures, and accessibility of research resources (e.g., policies, experts). (2) Institutional Regulatory Quality (3 items). This scale measures perceptions on how fair and respectful regulatory bodies (e.g., RECs, animal ethics committees) are with researchers and how familiar they are with the research they review. (3) Subunit Integrity Norms (4 items). This scale assesses perceptions on how much the department values scholarly integrity (e.g., honesty, confidentiality). (4) Subunit Integrity Socialization (4 items). This scale assesses perceptions of departmental commitment to socialize junior researchers in RCR effectively. (5) Subunit Advisor /Advisee Relations (3 items). This scale assesses perceptions about fairness, respect, and the availability of advisors to advisees. (6) Subunit Integrity Inhibitors (6 items). This scale measures respondents’ perceptions of the department’s negative impact on certain conditions (e.g., lack of adequate human or material resources, pressure to publish, competition among researchers). This scale is reversed-scored to express the lack of inhibitors. (7) Subunit Expectations (2 items). This scale assesses the perceptions related to the fairness of the departmental expectations for publishing and obtaining external funding.

The survey was delivered both in the original version, in English, and in the Hungarian translation. The English version was translated into Hungarian by two different translators. Then, a bilingual researcher familiar with the field of study cross-checked the translation, and the survey was back-translated into English. Any linguistic discrepancies were discussed until a satisfactory version was reached. Respondents could choose between English or the Hungarian version.

### Statistical Analysis

All the analyses were performed using IBM SPSS version 24.0. The mean scores for the seven subscales (RCR Resources, Regulatory Quality, Integrity Norms, Integrity Socialization, Advisor/Advisee Relations, Integrity Inhibitors, and Departamental Expectations) were calculated and stratified for academic rank and scientific field. The reliability coefficient was assessed by the Cronbach’s Alpha, where values > 0.70 express sufficient reliability or internal consistency. Measurements between groups and within groups were analyzed using the Kruskal-Wallis test, adjusted with Bonferroni correction. Multivariate regression analyses were performed. Multivariate regression analyses were performed to assess confounding interactions (with gender, age, academic rank, and scientific field) on the variables that were significant.

## Results

A total of 2557 people were invited to participate. 758 researchers opened the survey (30%). Of those who opened, 432 completed the survey (17%). Of the responding researchers, 46% were female, 54% were male, and two researchers did not disclose their gender. 44% are PhD students, 17% are postdocs or assistant professors, and 39% are an associate or full professors. 47% are from the Biomedical Sciences, 23% from Natural Sciences, 18% from Social Sciences, and 12% from Humanities.

Table 1. presents the number of cases, the mean scale score, standard deviation, the number of scales’ items, and the reliability coefficient. The Advisor/Advisee Relations scale had the highest score (3.92), Integrity Norms scored slightly lower (3.84), and Subunit Integrity Inhibitors scored the lowest (2.96). RCR Resources, Regulatory Quality, Integrity Socialization, and Departamental Expectations had average scores, ranging from 3.52 to 3.65. The reliability coefficient, expressed by Cronbach’s alpha (α), ranged from 0.69 to 0.88.

**Table 1:** Number of Cases, Mean Score, Standard Deviation, Number of items, and Reliability by scale

	RCR Resources	Regulatory Quality	Integrity Norms	Integrity Socialization	Advisor/ Advisee Relations	Integrity Inhibitors	Departamental Expectations
N	431	383	428	430	432	430	424
Mean	3.52	3.65	3.84	3.56	3.92	2.96	3.56
SD	0.93	0.91	0.85	0.88	0.86	0.82	0.96
Items	6	3	4	4	3	6	2
Cronbach’s alpha(α)	0.883	0.802	0.822	0.855	0.878	0.769	0.693

### Source Subscores and Academic Rank

For every scale, postdocs and assistant professors perceived integrity climate more negatively than PhD students and full or associate professors. In contrast, PhD students perceive more positively than the other groups (Table 2).

**Table 2:** Scales mean scores and standard deviation across academic rank

Academic rank	Ph.D. student	Postdoc or assistant professor	Associate or full professor	P*
RCR Resources	3.68 (0.96)	3.28 (0.86)	3.43 (0.89)	**0.00**
Regulatory Quality	3.83 (0.87)	3.50 (0.97)	3.51 (0.89)	**0.00**
Integrity Norms	3.99 (0.86)	3.71 (0.89)	3.73 (0.80)	**0.00**
Integrity Socialization	3.64 (0.95)	3.33 (0.85)	3.56 (0.80)	**0.01**
Advisor/Advisee relations	3.99 (0.93)	3.69 (0.91)	3.93 (0.73)	**0.01**
(Lack of) Inhibitors	3.01 (0.88)	2.91 (0.80)	2.92 (0.77)	0.55
Expectations	3.76 (0.97)	3.19 (0.87)	3.51 (0.94)	**0.00**

* Kruskall-Wallis test

When analyzed according to the academic rank groups, statistically significant differences were found for six subscales, Institutional RCR resources, Institutional Regulatory quality, Subunit Integrity Norms, Subunit Integrity Socialization, Advisor/Advisee Relations, and Subunit expectations.

Pairwise, adjusted using the Bonferroni correction, Institutional RCR resources scores were higher for PhD students (mean score = 3.68, 95% CI 3.54–3.81) when compared with postdocs and assistant professors (mean score = 3.28, 95% CI 3.08–3.48, P = 0.001), and to associate or full professors (mean score = 3.43, 95% CI 3.30–3.57, P = 0.014). There were no significant differences between the other pairs. In the Subunit Regulatory quality, PhD students scored higher (mean score = 3.83, 95% CI 3.70–3.97) when compared with postdocs and assistant professors (mean score = 3.50, 95% CI 3.25–3.74, P = 0.027), and to associate or full professors (mean score = 3.51, 95% CI 3.37–3.66, P = 0.002). In Integrity Norms subscale, PhD students scored higher (mean score = 3.99, 95% CI 3.87–4.11) when compared to postdocs and assistant professors (mean score = 3.71, 95% CI 3.50–3.92, P = 0.032), and to associate or full professors (mean score = 3.73, 95% CI 3.61–3.86, P = 0.006). There was no evidence of a difference between the other pairs. The PhD students’ scores (mean score = 3.64, 95% CI 3.50–3.77) in the Subunit Integrity Socialization were higher than postdocs or assistant professors (mean score = 3.33, 95% CI 3.14–3.53, P = 0.008). The PhD students’ scores (mean score = 3.99, 95% CI 3.86–4.12) were also higher in the Advisor /Advisee Relations subscale than postdocs or assistant professors (mean score = 3.69, 95% CI 3.48–3.90, P = 0.007). Subunit expectations scores were the lowest for postdocs or assistant professors (mean score = 3.19, 95% CI 2.99–3.39) when compared with highest, for PhD students (mean score = 3.76, 95% CI 3.62–3.90, P < 0.001), and with associate or full professors (mean score = 3.51, 95% CI 3.37–3.66, P = 0.040). PhD students also scored higher when compared with an associate or full professor (P = 0.032). Regulatory quality, Integrity Norms, and Advisor /Advisee Relations were confounded by the scientific field. However, when corrected for confounding with the scientific field, the associations remained significant (Supplementary 1).

### Source Subscores and Scientific Field

Overall, Natural sciences perceived integrity climate more negatively than the other scientific fields, and Humanities more positively (Table 3).

**Table 3:** Scales mean scores and standard deviation across scientific fields

Scientific field	Biomedical sciences	Natural sciences	Social sciences	Humanities	P*
RCR Resources	3.52 (0.97)	3.42 (0.89)	3.56 (0.96)	3.74 (0.84)	0.18
Regulatory Quality	3.79 (0.85)	3.49 (0.87)	3.62 (1.06)	3.80 (0.87)	**0.02**
Integrity Norms	3.79 (0.94)	3.75 (0.81)	3.98 (0.79)	4.10 (0.75)	**0.02**
Integrity Socialization	3.53 (0.95)	3.47 (0.80)	3.69 (0.88)	3.69 (0.94)	0.11
Advisor/Advisee relations	3.80 (0.94)	3.89 (0.81)	3.99 (0.85)	4.20 (0.71)	**0.04**
(Lack of) Inhibitors	3.09 (0.85)	2.87 (0.76)	2.81 (0.88)	3.12 (0.83)	**0.01**
Expectations	3.58 (0.90)	3.46 (0.93)	3.64 (1.10)	3.73 (0.95)	0.18

* Kruskall-Wallis test

The analysis of scientific fields provided statistically significant differences between scientific fields in four subscales: Regulatory quality, Integrity Norms, Advisor /Advisee Relations, and Inhibitors.

In pairwise analyses, Biomedical sciences (mean score = 3.78, 95% CI 3.64–3.93) scored significantly higher on Regulatory quality than Natural Sciences (mean score = 3.49, 95% CI 3.35–3.64, P = 0.025). Natural sciences score significantly lower (mean score = 3.75, 95% CI 3.62–3.87) on Integrity Norms than Humanities (mean score = 4.10, 95% CI 3.88–4.31, P = 0.032). Humanities (mean score = 4.20, 95% CI 4.00–4.40) also scored significantly higher than Biomedical sciences (mean score = 3.80, 95% CI 3.64–3.96) on Advisor /Advisee Relations (P = 0.046). There was no evidence of a difference between the other pairs. When corrected for confounding with the academic rank, the associations remained significant (Supplementary 2).

## Discussion

This study aimed to assess Hungary’s integrity climate and the differences across scientific fields and academic rank using the SOURCE questionnaire. To our knowledge, this is the first study to investigate the research climate in Hungarian institutions.

Overall, the study results highlight some critical points of the integrity climate in Hungary. The study findings show that overall mean scores for all scales were below 4.00. The results contrast with the findings from the U.S. by Martinson et al., (2016), where most results were above 4.00. The results were also lower across all scales compared to the study by Wells et al., (2014) and in most of the scales compared to those found by Crain et al., (2013). Although comparable to the results from Amsterdam by Haven et al., (2019), with the results falling between 3.20 and 3.80, the patterns differed across scales.

The scales Advisor/Advisee relations and Integrity norms had the highest scores in the study, while (the lack of) Integrity Inhibitors scored the lowest. Thus, although study participants perceive more positively the attitude of supervisors and the support of norms, they are exposed to different factors of Integrity Inhibitors. The integrity inhibitors express the presence of certain conditions, such as lack of resources (human or material), pressure to obtain funding, pressure to publish, suspicion, and competition among researchers. Publication pressure and competition among researchers can be harmful to the integrity of science. These factors affect the willingness to open sharing of information, interfere with the peer-review process, and lead to detrimental research practices (Anderson et al., 2007; Fanelli, 2010). The Hungarian findings had the worst scores on this scale, contrasting with the findings from The Netherlands (Haven et al., 2019) and the U.S. (Wells et al., 2014). This might be explained by similarities in competitiveness and pressure to publish trends in academia that are not accompanied by similar human and material resources at these research institutions. Researchers at the studied universities often have to balance their research activities with teaching duties. Moreover, Hungarian universities have much more modest financial resources for scientific research than universities in the U.S. or the Netherlands.

Academic rank has shown to be significantly related to six scales. Surprisingly, the mean patterns by ranks were identical across all scales. Overall, PhD students perceive more positively than the other groups, while postdocs and assistant professors perceive more negatively. These results are similar to those found by Wells et al., (2014) in the U.S., and contrast to the results found by Haven et al., (2019) in Amsterdam, where PhD students scored lower than professors across all scales. The potential explanations by Wells et al., (2014) for the low scores of postdocs and assistant professors were shorter time spent in the research environment and less (familiarity) contact with the institution and its functions. Recent surveys (Afonja et al., 2021; Woolston, 2020a, b) show that postdocs are exposed to higher pressures to publish and obtain funding than PhD students due to an unstable work position and a competitive environment. The little job security exposes them to higher expectations to secure their position. The surveys have also shown that postdocs in their academic position get little or no guidance or resources for their work. These findings could explain the lowest scores of postdocs and assistant professors.

The scientific field was also significantly related to perceived integrity climate in four scales. However, the mean patterns by disciplines are not similar across all scales, except Humanities, which had the highest scores for every scale. The Humanities results contrast with the results found by Haven et al., (2019), where Humanities scored lowest in most of the scales. The high scores of the Humanities could be explained by their separateness from the research system of the more empirical and quantitative scientific fields, especially fields like biomedical research. In Hungarian universities, Humanities still focus on books as the major research output. International competitiveness is less present, and some of the research integrity’s challenges (e.g., Projects’ submission to RECs) are absent from their everyday work. In Regulatory quality, pairwise differences were found between Biomedical and Natural sciences. A higher score for Biomedical sciences is expected as regulatory bodies such as research and animal ethics committees are essential to biomedical research. The same might not apply to natural sciences disciplines, such as Mathematics or Physical sciences.

In the Integrity norms scale, researchers from Humanities perceive how much the department values scholarly integrity more positively than those from Natural Sciences. In Advisor/Advisee Relations, Humanities scored higher than Biomedical sciences. Recent evidence highlights ethical issues involving mentoring in Medical Sciences, such as misalignment of goals, poor communication, and failure to acknowledge the advisee’s contribution (Kow et al., 2020). However, little is known about this context in Humanities. One potential explanation could refer to the differences regarding the disciplinary traditions of mentoring in PhD student-supervisor or Postdoc trainee-supervisor relationships in the Humanities.

### Limitations

Our study has some limitations that should be addressed. First, there was low interest in participation by Doctoral Schools. Only 24% of the invited Doctoral Schools actively agreed to participate by sending us feedback. This might demonstrate a low interest of Doctoral Schools in investigating institutional integrity climate. Although the study was completely anonymous and there was no comparison between units or departments, fear of ranking or retaliation is a potential explanation, as the results can be sensitive. Consequently, there is a possibility that the Doctoral Schools that have agreed to participate are the ones that most foster integrity, which creates a selection bias.

Moreover, the response (30%) and completion (17%) rates were relatively low. However, the rates are comparable to other web-based surveys (Cook et al., 2000; Haven et al., 2019). It is challenging to determine the reason for the low response rate. The study was conducted amid the COVID-19 pandemic, where academics often had to balance their responsibilities and domestic activities. Therefore, the reseachers’ decision to participate in the survey might have differed.

Second, as there was no data collection regarding departments or programs to ensure anonymity, it was not possible to investigate differences between sub-units. In the results by Wells et al., (2014), differences across small organizational units account for a great part of the variability in the scales. (Cook et al., 2000; Haven et al., 2019)

## Conclusions

Research integrity climate is a strong factor that influences an individual’s behavior. Negative perceptions of the research climate were associated with a higher likelihood of engaging in detrimental research practices (Crain et al., 2013). Hence, a strong research integrity culture can lead to better research practices and responsible conduct of research (Forsberg et al., 2018). While everyone involved in the research endeavor is responsible for the ethics and integrity of science, there has been a call for research performing organizations to provide measures to strengthen integrity (Forsberg et al., 2018; Mejlgaard et al., 2020).

Institutional initiatives for promoting and fostering integrity should be evidence-supported and as tailored as possible (Viđak et al., 2021). Organizational climate investigations are a valuable instrument to identify the strengths and weaknesses of each subgroup to develop targeted initiatives and institutional policies. Our study indicated some critical points regarding the integrity climate in Hungarian universities. If we look at scientific fields, overall, Natural sciences perceived integrity climate more negatively than other fields. Therefore, department leaders should develop initiatives to address the weaknesses and foster better integrity climates. It is essential to provide clear guidelines and policies on RCR and keep an open dialogue on RI at the institutional and departmental levels. According to academic ranks, postdocs and assistant professors perceived the integrity climate more negatively on every scale. The results suggest that institutions should pay more attention to early career researchers, especially those in insecure and transitory work positions. They should provide RCR resources, socialize them in RCR, and set more reasonable expectations.

The low scores on integrity inhibitors indicate that responsible research conduct is impacted by non-ideal research conditions related to resources, pressure, and interpersonal relations. These factors require further detailed studies developed for these institutional contexts.

## Electronic Supplementary Material

Supplementary material 1

Supplementary material 2

## Data Availability

Data cannot be shared publicly due to participants’ privacy. The pseudonymized data might be made available under a data transfer agreement for research purposes only. The research protocol is available at https://osf.io/n92z3/?view_only=72a073ebdb9c4f1992fd259b2ebb733b.
