# Supplementary material for: Perceptions of Research Integrity Climate in Hungarian Universities: Results from A Survey among Academic Researchers
Source: Sci Eng Ethics. 2022 Jun 30;28(4):30. doi: 10.1007/s11948-022-00382-5 (PMC9245862; doi:10.1007/s11948-022-00382-5)
Supplement: Supplementary file 1 — Supplementary material 1 [file 11948_2022_382_MOESM1_ESM.doc]

| Academic rank | Postdoc | | Professor | | PhD student | |
| --- | --- | --- | --- | --- | --- | --- |
| Subscale *F (p,df)* | Beta | (CI) | Beta | (CI) | Ref | Ref |
| RCR resources  6.188 (.002, 2) | -.161 | (-.644,-.148) | -.123 | (-.431,-.040) | - | - |
| Regulatory Quality  6.177 (.002,2) | -.143 | (-.614,-.091) | -.180 | (-.532,-.131) | - | - |
| Integrity Norms  5.193 (.006,2) | -.116 | (-.491,-.033) | -.128 | (-.404,-.045) | - | - |
| Integrity Socialization  3.227 (.041, 2) | -.121 | (-.519,-.043) | -.022 | (-.228,.147) | - | - |
| Advisor/Advisee relations  3.432 (.003,2) | -.129 | (-.523,-.063) | -.014 | (-.206,.155) | - | - |
| Expectations  9.941 (<.001,2) | -.221 | (-.819,-.304) | -.120 | (-.439,.034) | - | - |

Supplementary 1. Regression models of SOURCE subscales and academic rank.

F-tests (*F*) and the associated p-value and degrees of freedom. Regression coefficients (Beta) and confidence intervals (CI) adjusted for confounding factor (scientific field).
