# Supplementary material for: Perceptions of Research Integrity Climate in Hungarian Universities: Results from A Survey among Academic Researchers
Source: Sci Eng Ethics. 2022 Jun 30;28(4):30. doi: 10.1007/s11948-022-00382-5 (PMC9245862; doi:10.1007/s11948-022-00382-5)
Supplement: Supplementary file 2 — Supplementary material 2 [file 11948_2022_382_MOESM2_ESM.doc]

Supplementary 2. Regression models of SOURCE subscales and scientific field.

| Scientific field | Natural Sciences | | Social Sciences | | Humanities | | Biomedical Sciences | |
| --- | --- | --- | --- | --- | --- | --- | --- | --- |
| Subscale  *F (p,df)* | Beta | (CI) | Beta | (CI) | Beta | (CI) | Beta | (CI) |
| Regulatory Quality  2.772 (.041,3) | -.153 | (-.500,-.075) | -.110 | (-.517, .006) | -.006 | (-.334, .300) | Ref | - |
| Integrity Norms  3.013 (.030,3) | -.186 | (-.592,-.058) | -.070 | (-.455, .147) | Ref | - | -.156 | (-.558,-.011) |
| Advisor/Advisee relations  2.946 (.033,3) | -.172 | (-.571,-.035) | -.103 | (-.528, .073) | Ref | - | -.219 | (-.678,-.129) |

F-tests (*F*) and the associated p-value and degrees of freedom. Regression coefficients (Beta) and confidence intervals (CI) adjusted for confounding factors (academic rank).
